# MI-DenseCFNet: deep learning–based multimodal diagnosis models for *Aureus* and *Aspergillus* pneumonia

**DOI:** 10.1007/s00330-023-10578-3

**Published:** 2024-01-17

**Authors:** Tong Liu, Zheng-hua Zhang, Qi-hao Zhou, Qing-zhao Cheng, Yue Yang, Jia-shu Li, Xue-mei Zhang, Jian-qing Zhang

**Affiliations:** 1 The Second Department of Respiratory and Critical Care Medicine, The First Affiliated Hospital of Kunming Medical University, No. 295, Xichang Road, Wuhua District, Kunming, Yunnan 650032 People’s Republic of China; 2 Medical Imaging Department, The First Affiliated Hospital of Kunming Medical University, Kunming, Yunnan 650032 People’s Republic of China; 3 School of Information, Yunnan University, Kunming, Yunnan 650032 People’s Republic of China

**Keywords:** Communicable diseases, Pneumonia, Deep learning, Artificial intelligence

## Abstract

**Objective:**

To build and merge a diagnostic model called multi-input DenseNet fused with clinical features (MI-DenseCFNet) for discriminating between *Staphylococcus aureus* pneumonia (SAP) and *Aspergillus* pneumonia (ASP) and to evaluate the significant correlation of each clinical feature in determining these two types of pneumonia using a random forest dichotomous diagnosis model. This will enhance diagnostic accuracy and efficiency in distinguishing between SAP and ASP.

**Methods:**

In this study, 60 patients with clinically confirmed SAP and ASP, who were admitted to four large tertiary hospitals in Kunming, China, were included. Thoracic high-resolution CT lung windows of all patients were extracted from the picture archiving and communication system, and the corresponding clinical data of each patient were collected.

**Results:**

The MI-DenseCFNet diagnosis model demonstrates an internal validation set with an area under the curve (AUC) of 0.92. Its external validation set demonstrates an AUC of 0.83. The model requires only 10.24s to generate a categorical diagnosis and produce results from 20 cases of data. Compared with high-, mid-, and low-ranking radiologists, the model achieves accuracies of 78% vs. 75% vs. 60% vs. 40%. Eleven significant clinical features were screened by the random forest dichotomous diagnosis model.

**Conclusion:**

The MI-DenseCFNet multimodal diagnosis model can effectively diagnose SAP and ASP, and its diagnostic performance significantly exceeds that of junior radiologists. The 11 important clinical features were screened in the constructed random forest dichotomous diagnostic model, providing a reference for clinicians.

**Clinical relevance statement:**

MI-DenseCFNet could provide diagnostic assistance for primary hospitals that do not have advanced radiologists, enabling patients with suspected infections like *Staphylococcus aureus* pneumonia or *Aspergillus* pneumonia to receive a quicker diagnosis and cut down on the abuse of antibiotics.

**Key points:**

*• MI-DenseCFNet combines deep learning neural networks with crucial clinical features to discern between Staphylococcus aureus pneumonia and Aspergillus pneumonia.*

*• The comprehensive group had an area under the curve of 0.92, surpassing the proficiency of junior radiologists.*

*• This model can enhance a primary radiologist’s diagnostic capacity.*

**Supplementary Information:**

The online version contains supplementary material available at 10.1007/s00330-023-10578-3.

## Introduction

According to the Global Burden of Disease 2019 study, more than 2.49 million deaths are caused by lower respiratory tract infections, including pneumonia and fine bronchitis [1, 2]. Identifying pneumonia pathogens is challenging and time-consuming, but the imaging results obtained from computer tomography (CT) scans can offer a certain level of characterization for pulmonary pathological changes corresponding to specific pathogenic microorganisms. This allows for improved quantification and measurement of lesion size and degree or severity of lung involvement, potentially leading to faster diagnosis compared to molecular diagnosis in the laboratory [3]. However, the interpretation of imaging histology involves visual perception and uncertain judgmental decisions, inevitably resulting in human misinterpretation of image results [4].

Automated review and simultaneous analysis of numerous quantitative features of CT images and their degree of correlation are enabled by artificial intelligence [5]. Conversely, deep learning neural networks under artificial intelligence can automatically learn features from image data without the need for human predefinition [6, 7]. However, there are few studies on the combination of clinical features with image-assisted diagnostic models, and combining these models can result in a more accurate diagnosis.

Certain specific imaging features (such as air crescent sign, air sacs, and halo sign) distinguish pneumonia caused by *Staphylococcus aureus* pneumonia (SAP) and *Aspergillus* pneumonia (ASP), but there are also similar imaging features (such as cavities, exudates, solids, and nodules) that cannot be distinguished with great precision by inexperienced physicians [8, 9]. Given the distinct treatment options and prognosis associated with lung infections caused by these two pathogens, delayed identification of the pathogen could result in unfavorable outcomes due to empirical drug use alone [10, 11]. Combining imaging-assisted diagnostic models with clinical features to achieve early predictions of SAP and ASP can significantly aid in making clinical treatment decisions.

Thus, this study aimed to develop and validate the MI-DenseCFNet multimodal diagnostic model using a deep learning neural network. Furthermore, the study aims to identify clinical features significantly associated with SAP and ASP using a random forest binary diagnostic model, which combines standard machine learning (ML) with patients’ clinical data to provide a reference for clinicians to aid in making treatment decisions.

## Patients and methods

### Patient cohort and data collection

**Fig. 1:**
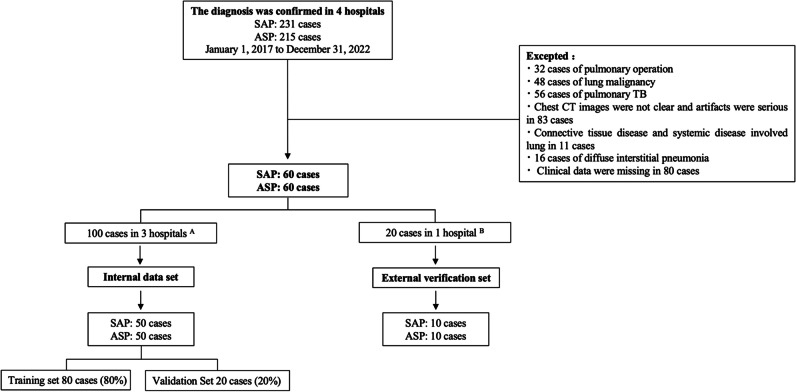
^A^The first affiliated hospital of kunming medical university, the first people’s hospital of yunnan province, and the kunming yan’an hospital; ^B^Kunming first people’s hospital ganmei hospital

This study was conducted in accordance with the relevant guidelines and regulations. According to Chinese legislation and system requirements, the ethical review committee of the First Affiliated Hospital of Kunming Medical University approved and exempted written informed consent for this retrospective study. A total of 231 clinically confirmed SAP and 215 ASP were selected from four large tertiary hospitals in Kunming, China. After the exclusion of ineligible patients by two chief radiologists and a chief physician who specialized in respiratory infections according to the inclusion and exclusion criteria, a final selection of 60 patients with SAP and 60 patients with ASP was achieved. From the picture archiving and communication system, 31,259 high-resolution CT lung windows of the chest of all patients were exported, and the clinical information of the corresponding patients was collected from the electronic medical record database of each hospital (Fig. 1).

Inclusion criteria include the following: (1) the diagnostic criteria for patients as per the relevant guidelines of the American Thoracic Society[12]; (2) identifying pathogens by deep sputum culture, blood culture, bronchoalveolar lavage culture, histopathology, and macrogenomic sequencing, with the above identification serving as the “gold standard” for the pathogenesis of SAP and ASP.

Exclusion criteria: (1) patients with heart failure; (2) patients with pulmonary surgery history; (3) individuals presenting diffuse interstitial lung lesion on chest CT; (4) individuals affected by connective tissue disease or systemic disease involving the lungs; (5) patients with pulmonary malignancy, active tuberculosis, or pulmonary embolism; (6) cases where chest CT images are unclear or pseudo-shadows are obvious and cannot meet the requirements of diagnosis and analysis; (7) instances with inadequate clinical information.

### Image data

#### Collection of CT images

All patients were scanned using a GE 64-row CT machine, with parameters set to 100–130 kV tube voltage, automatic tube current modulation, 192 × 0.6 mm or 128 × 0.625 mm detector collimation, and 1 to 2.5 mm slice thickness. One of the following CT scanners was used to examine all patients: Brilliance iCT, GE Discovery CT 750 HD, Somatom Definition, Somatom Emotion and IQon Spectral. A high kernel (b60) and a 512 × 512 matrix were employed for reconstructing all images, and image data were exported in the Dicom format.

#### Preprocessing of CT Images

Mimics Research 19.0 software converted the collected image data from the Dicom format to BMP format files, resulting in an export of 13,964 images after removing slices without image features. To meet the training backbone requirements, data enhancement was performed through operations such as horizontal flipping, random scaling, and random panning. Furthermore, data for each image were normalized to 0 and 1. This operation is to standardize the pixel value of the image to the range of 0 to 1, where 0 represents black and 1 represents white. It is a common preprocessing step in training neural networks, which can stabilize the training process, accelerate the convergence rate, and reduce the model’s dependence on specific attributes of the image.

### Construction of MI-DenseCFNet multimodal diagnosis models

**Fig. 2:**
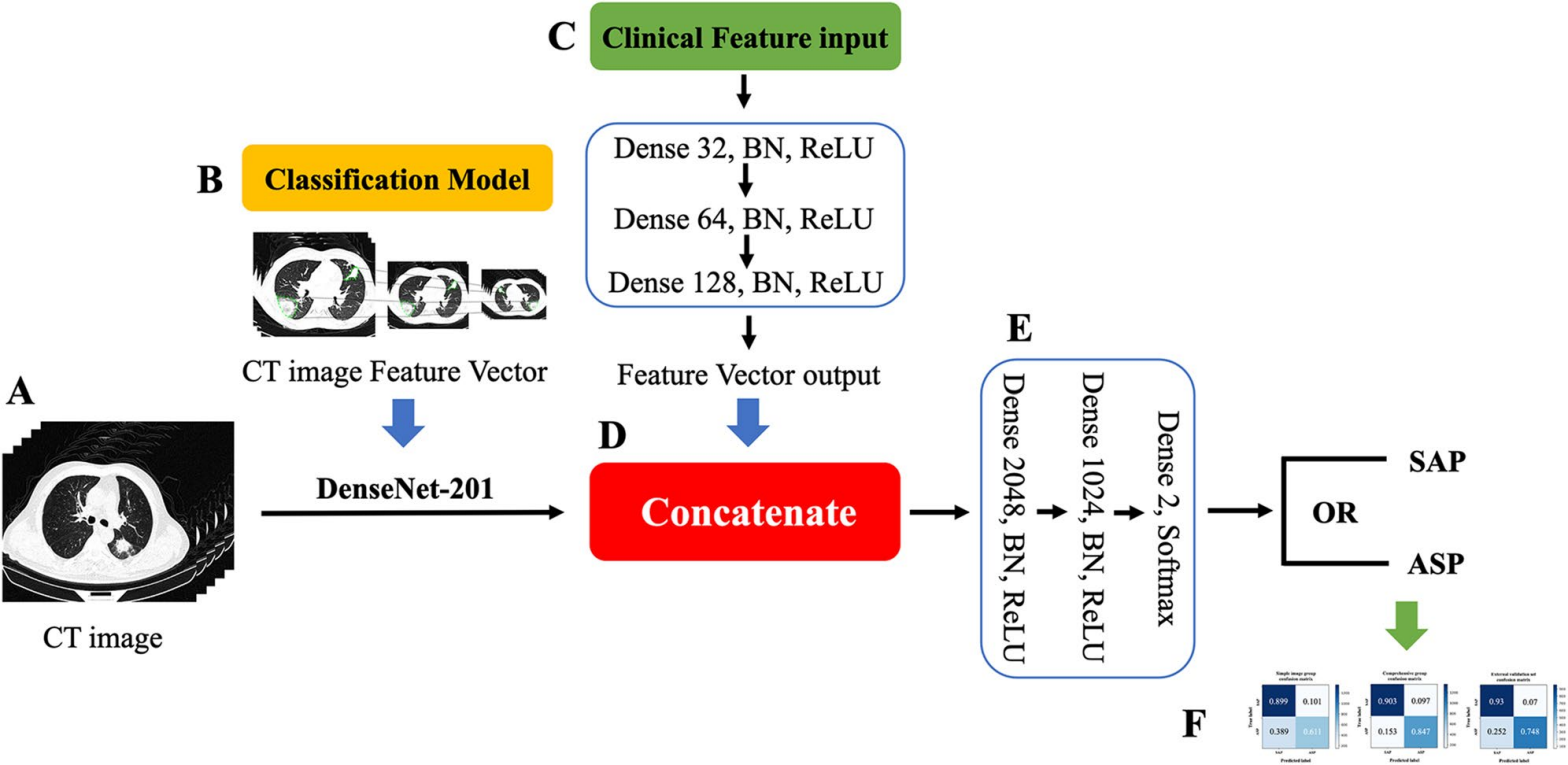
(**A**) Preprocessed CT image set; (**B**) Skeleton network DenseNet-201 for extracting feature vectors from CT image sets; (**C**) Input of clinical information (clinical symptoms, laboratory results, imaging features) is fed into a three-layer deep neural network (DNN) for training and extracting clinical feature vectors; (**D**) Converging and connecting clinical and image feature vectors; (**E**) After fusing clinical and image feature vector information, it is fed into a DNN with two layers for training, and finally, a DNN with two neurons outputs the classification and diagnosis results; (**F**) Confusion matrix visualization classification diagnosis results

MI-DenseCFNet multimodal diagnostic model, a fusion model based on a deep learning neural network, comprises two modules: a deep learning imaging histology classification diagnostic model and a deep neural network (DNN) clinical feature extraction model. Figure 2 shows the schematic of the model.

#### Construction of the deep learning imaging histology classification and diagnosis model

To extend the training data, enhance the model generalization, and prevent overfitting, migration learning fine-tuning was employed [13]. A total of 135,609 single-channel gray CT images corresponding to 2719 patients were sourced from the China National Center for Bioinformation (CNCB) website, and invalid slices were censored for migration training of the model. This approach enables the DenseNet-201 deep learning model to grasp the basic features of chest CT images in advance (CNCB website:https://www.cncb.ac.cn).

Subsequently, the weights of the classification model DenseNet-201 after data extension learning were loaded into the DenseNet-201 model for SAP and ASP chest CT imaging tasks. The training set data were employed to develop the model, whereas the validation set data were used for independent testing of the model and to compare the internal validation set data with the diagnostic performance of radiologists (Supplemental 1). Then, accuracy, precision, sensitivity, specificity, and F1 score were calculated based on the classification and diagnosis results produced by the deep learning algorithm. The receiver operator characteristic curve (ROC) was plotted using the matplotlib package in Python (version: 3.8.12, http://www.Python.org). To evaluate the consistency of diagnostic performance, Kappa values were computed using the scikit-learn package, with Kappa values < 0.40, 0.40–0.75, and > 0.75 indicating low, moderate, and excellent consistency, respectively. To plot the heat map of the model, gradient-weighted class activation mapping was employed. Gradient-weighted Class Activation Mapping (Grad-CAM) [14] was used for thermal map visualization to further verify the effectiveness of the method. Grad-CAM combines overall situation averaging pooling and weighting of gradient signals to generate heat maps that highlight areas of the image that have a significant impact on the final prediction results. It does this by calculating the gradient of the final convolutional layer to determine which feature maps in that layer are important for the classification of a particular class. By multiplying and summing these gradients with the feature map, followed by ReLU activation and global averaging pooling, Grad-CAM produces a heat map that shows the regions of the image associated with the predicted results.

#### Deep neural network clinical feature extraction model

To further enhance the diagnostic accuracy of the model, we employed a DNN to extract clinical features. The clinical data used includes structured data from hospital records (such as patient age, gender, medical history, and text descriptions from image reports), all entered into the DNN in digital form and combined with the raw image data to provide more comprehensive information to the DNN.

Since the clinical data employed in this study are categorized into clinical symptoms, laboratory results, and imaging features (comprising 49 features, including demographic features), three 3-layer DNNs were employed to extract features for each of these three types of data (Fig. 2)

#### Mathematical logic of MI-DenseCFNet model

The CT image feature vectors ***V***_*bone*_ ∈ ***R***^1× *C*^ (C represents the dimension of the eigenvector) extracted by the skeleton network and the clinical feature vectors extracted by three deep neural networks with the same structure (image feature vectors ***V***_*img*_ ∈ ***R***^1×128^, clinical symptom feature vectors ***V***_*clic*_ ∈ ***R***^1×128^, and laboratory examination indicator feature vectors ***V***_*lab*_ ∈ ***R***^1×128^) are concatenated along the dimension axis to form vector ***V*** ∈ ***R***^1×  (*C*  +128×3)^. Then, ***V*** is fed into a fully connected network composed of 2048, 1024, and 2 neurons to learn these Homologous isomerism data. Finally, a binary probability vector is output through the Softmax activation function (Fig. 2)

In this study, Softmax is chosen as the activation function of binary classification instead of Sigmoid because Sigmoid and Softmax are theoretically equivalent in binary classification problem, and the function expression is as follows:

1-1 $$Sigmoid\left({x}_1\right)=\frac{1}{1+{e}^{-{x}_1}}$$

1-2 $$Softmax\left({x}_1\right)=\frac{e^{x_1}}{e^{-{x}_1}+{e}^{-{x}_2}}=\frac{1}{1+{e}^{-\left({x}_1-{x}_2\right)}}$$

where *x*_1_ and *x*_2_ are inputs, *x*_1_ is selected as the positive example of input this time, and it can be seen from the formula (1-2) that (*x*_1_-*x*_2_) can be replaced by *z*_1_, that is, Softmax(x1) can be written as:

1-3 $$Softmax\left({z}_1\right)=\frac{1}{1+{e}^{-{z}_1}}$$

From Eqs. (1-1) and (1-3), it can be seen that when the Softmax function has only two inputs, it is actually the Sigmoid function, which is completely equivalent in binary classifications. Therefore, we use Softmax as the activation function for the probability output in this binary classification problem.

#### Emphasize and illustrate

DenseNet-201 is a neural network model for learning standardized CT images. It mainly learns some texture features on the images, which may not be recognized by the human eye. In the DNN clinical feature extraction model, radiologists first read the CT images of the included patients and diagnosed the corresponding clinical image features (this is a conclusive report), and then these structured data were input into the DNN in the form of numbers.

- *Similarities*: Both models are trained on the same set of patients, and the images correspond to the clinical data.
- *Differences*: The input data of DenseNet-201 is standardized CT images, and the input data of DNN is structured clinical data.

### Construction of a machine learning classification and diagnosis system

The construction of the random forest dichotomous diagnosis model was completed using RStudio (version 2022.12.0-353) software equipped with R language (version 4.2.2).

#### Random forest dichotomous models

Caret and random Forest packages were employed to construct the disease classification and diagnosis model. First, the create Data Partition function in the caret package was used to randomly divide the clinical features of the internal dataset into training and validation sets at a ratio of 8:2. An initial screening of the 49 included features (encompassing demographic features, clinical symptoms, laboratory findings, and imaging features) was performed using the recursive feature elimination method. Subsequently, the random forest algorithm was employed to further filter out significant classification features using the following parameter settings: mtry = 3 and ntree = 500. The selection of the top-scoring variables was based on the variable importance score indicators Mean Decrease Accuracy and Mean Decrease Gini. The most important classification features were derived by identifying their intersections. To plot random forest ROC curves, the pROC package was loaded, and the diagnostic classification performance of the model was quantitatively assessed based on the ROC curves.

## Statistical methods

SPSS 26.0 was used to analyze the differences between the clinical characteristics of SAP and ASP. A *p* value < 0.05 was considered a statistically significant difference.

## Results

### Characteristics of the study population

In this study, 120 patients were included, comprising 60 patients with SAP and 60 patients with ASP. Among these patients, 70 were male and 50 were female, with 51 having a history of smoking and 69 without a history of smoking. The age range of the participants was 7–82 years, with a median age of 50 years. The study identified 27 cases in the age group > 60 years and 93 cases in the age group ≤ 60 years. Employing an independent sample t-test, it was concluded that no statistical differences existed between patients with SAP and ASP in terms of gender, age, and history of smoking, as indicated by *p* value > 0.05 (Table 1).

**Table 1:** Demographic characteristics of the enrolled patients

Clinical factors	ASP (*n* = 60)	SAP (*n* = 60)	*p*
Gender *n *(%)			0.065
Male	30 (50%)	40 (66.7%)	
Female	30 (50%)	20 (33.3%)	
Age (year) *n *(%)			0.36
> 60	12 (20%)	15 (25%)	
≤ 60	48 (80%)	45 (75%)	
Smoking history *n *(%)			0.516
Smoking	23 (38.3%)	28 (46.7%)	
Non-smoking	37 (61.7%)	32 (53.3%)	
Past history *n *(%)			
Diabetes	10 (16.7%)	13 (21.7%)	
Hypertension	3 (5%)	13 (21.7%)	
Gout	2 (3.3%)	4 (6.7%)	
Leukemia	1 (1.7%)	1 (1.7%)	
Renal failure	1 (1.7%)	3 (5%)	
SLE	2 (3.3%)	2 (3.3%)	
Hyperthyroidism	1 (1.7%)	0	
Oral glucocorticoids	2 (3.3%)	1 (1.7%)	
Immunosuppression	2 (3.3%)	0	
Postoperative*	0	6 (10%)	

*SLE*: systemic lupus erythematosus; * for postoperative patients except for lung surgery (brain surgery, debridement, etc.)

### Internal validation of the MI-DenseCFNet multimodal diagnostic model

Generally speaking, the training of neural networks is a process of continuous optimization results; that is, with the increase in the number of training rounds, the training index will become better and better and constantly close to the ideal state. Therefore, the training result of the neural network is not a process worthy of attention, but the result of the verification set that is not trained.

**Fig. 3:**
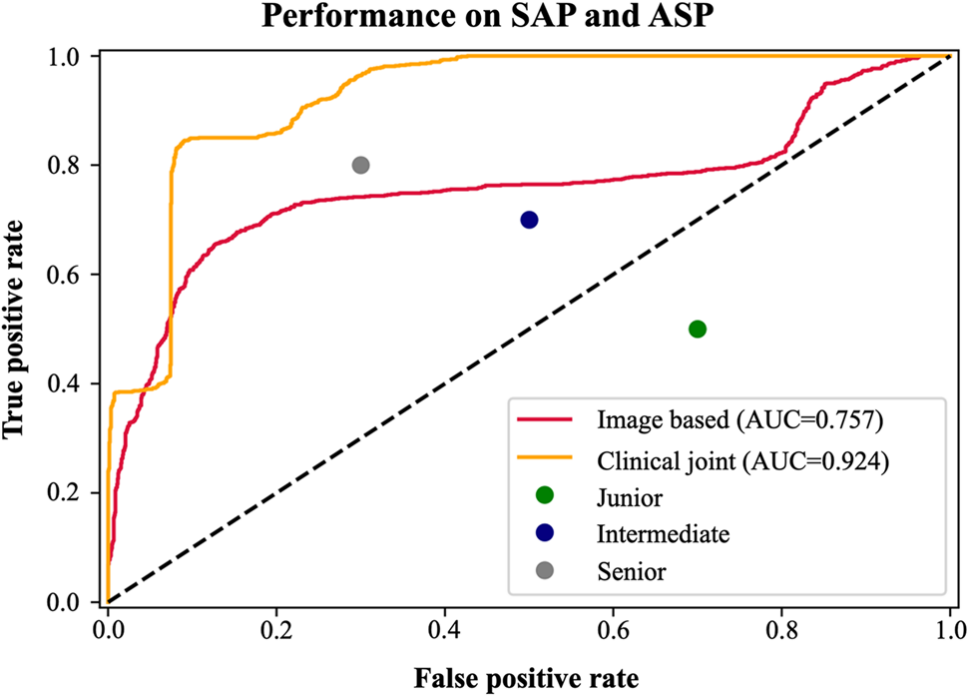
Image-based: Image-only group model [DenseNet-201]; Clinical Joint: Integrated group (clinical+imaging) model [MI-DenseCFNet]

**Fig. 4:**
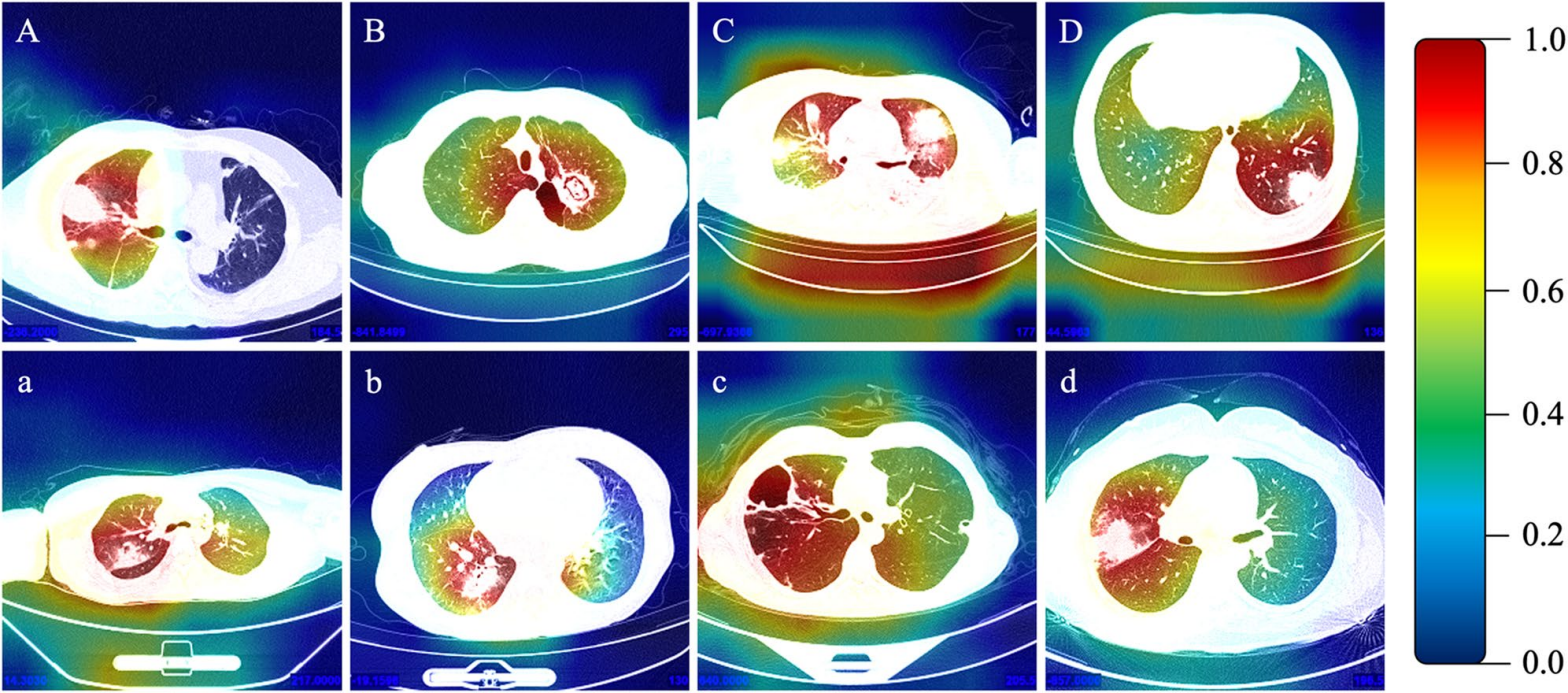
**A**–**D** and **a**–**d** are the CT images of suspicious lesion areas detected by the deep learning diagnostic model for ASP and SAP, respectively. The color bar on the right panel indicates the intensity of attention, with a darker red color indicating the strongest attention and a darker blue color indicating weaker attention

The imaging-only group model [DenseNet-201] exhibited an area under the curve (AUC) of 0.76, with an accuracy of 78.4%, precision of 78.8%, sensitivity of 75.5%, specificity of 75.5%, F1 score of 0.771, and Kappa value of 0.53 (Fig. 3, Supplemental 2). The classification and diagnosis efficiency consistency of this model was moderate. After adding the DNN model to extract clinical features, the integrated group (clinical + imaging) model [MI-DenseCFNet] demonstrated an AUC of 0.92, accuracy of 88.1%, precision of 87.6%, sensitivity of 87.5%, specificity of 85.3%, F1 score of 0.875, and Kappa value of 0.75 (Fig. 3, Supplemental 2). The classification and diagnosis efficiency consistency of this model was excellent. Figure 4 illustrates the heat map of the model.

### Performance verification

#### Comparison of the radiologist and model performance

The DenseNet-201 model required 10.24 s for diagnosis, with an accuracy rate of 78%. The low-ranking physician employed 1178.56 s for diagnosis, with an accuracy rate of 40%. Similarly, the middle-ranking physician took 626.37 s for diagnosis, with an accuracy rate of 60%. The high-ranking physician took 463.28 s for diagnosis, with an accuracy rate of 75% (Supplemental 3).

#### External validation

**Fig. 5:**
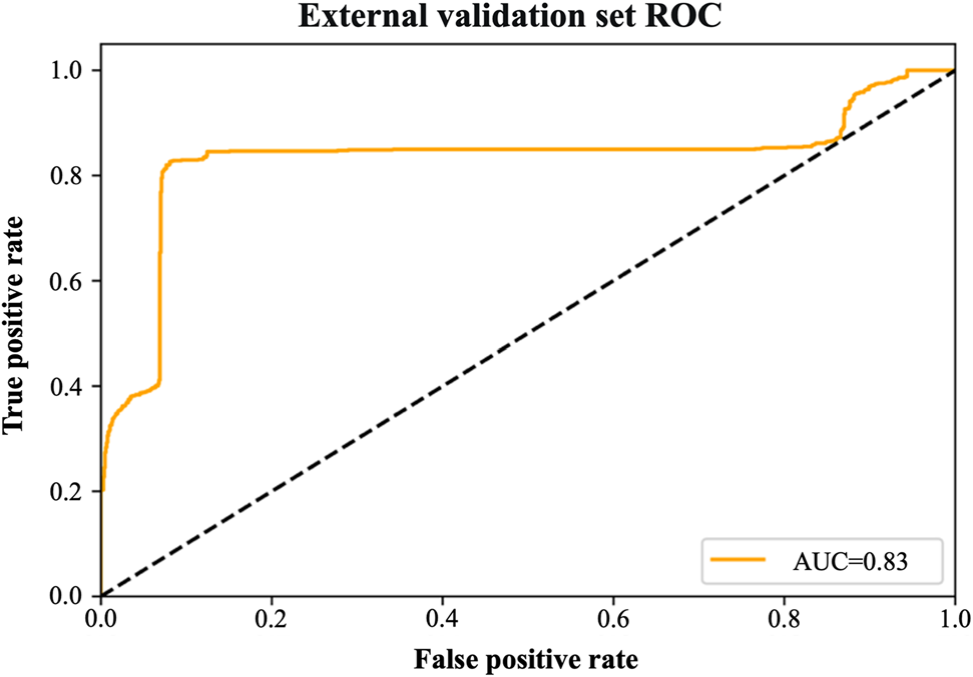
External validation set ROC curve

For validation, chest CT images and clinical information from the external validation set were imported into the MI-DenseCFNet diagnostic model. The external validation set exhibited an AUC of 0.83, diagnostic accuracy of 84.3%, precision of 85.4%, sensitivity of 83.9%, specificity of 83.9%, F1 score of 0.846, and Kappa value of 0.68 (Fig. 5 and Supplemental 2). Furthermore, the external validation set demonstrated a moderate consistency of classification and diagnosis efficiency.

### Classification of SAP and ASP by the model

**Fig. 6:**
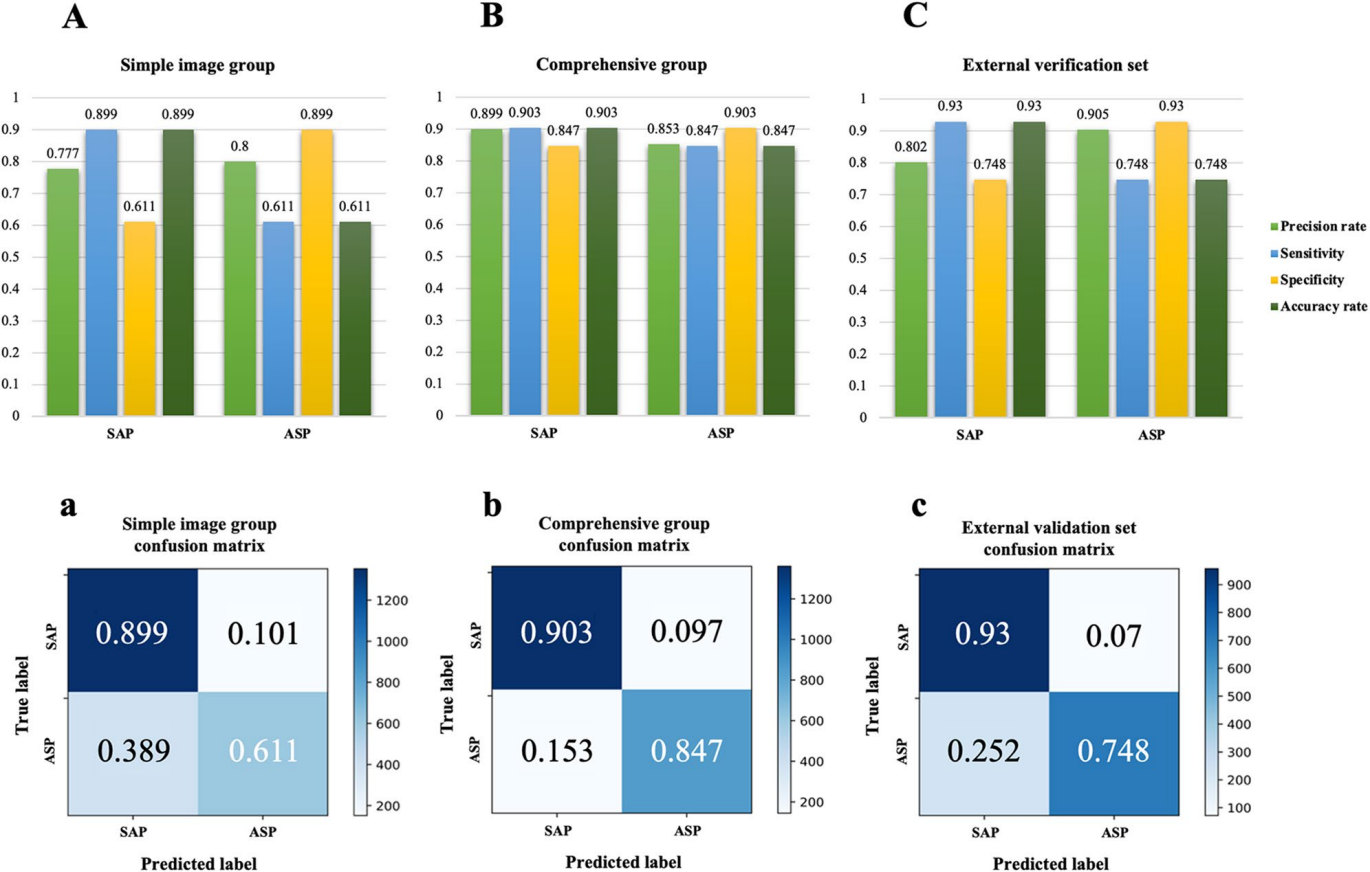
**A–C** Model diagnostic performance cases of ASP and SAP; **a**–**c** confusion matrix

An analysis of the diagnostic accuracy of SAP and ASP indicated accuracy rates of 89.9% for SAP and 61.1% for ASP in the imaging-only group (Fig. 6A). In the combined group, the corresponding accuracy was 90.3% for SAP and 84.7% for ASP (Fig. 6B). Furthermore, in the external validation group, SAP and ASP exhibited diagnostic accuracy rates of 93% and 74.8%, respectively (Fig. 6C).

### Random forest classification diagnosis model based on machine learning

Based on the variable importance score index, 20 variables were finally selected (Supplemental 4). After taking the intersection set, 11 important classification features were filtered out (Table 2). Using these 11 important classification features, a random forest classification diagnostic model was developed in the training set. Consequently, the AUC for the model in the training set was 0.812 (95% Cl, 0.726–0.899), whereas that in the validation set was 0.95 (95% Cl, 0.852–1) (Supplemental 5).

**Table 2:** Statistical tests of the variables

Name	ASP (*N* = 50)	SAP (*N* = 50)	*p*
GM test	1.6 ± 2.6	0.1 ± 0.1	< 0.001
Halo sign	1.6 ± 0.5	2.0 ± 0.0	< 0.001
Air crescent sign	1.6 ± 0.5	2.0 ± 0.0	< 0.001
Albumin (g/L)	34.4 ± 7.6	29.9 ± 6.5	0.002
CRP (mg/L)	57.5 ± 90.7	111.7 ± 109.4	0.008
Neutrophils (× 10^9^/L)	7.1 ± 6.1	11.6 ± 12.6	0.03
G test (pg/mL)	68.6 ± 120.1	26.0 ± 74.4	0.036
WBC (× 10^9^/L)	9.5 ± 6.4	11.9 ± 5.6	0.049
PCT (ng/mL)	1.2 ± 4.9	9.0 ± 29.3	0.072
Lactic acid (mmol/L)	1.8 ± 1.3	1.5 ± 1.1	0.196
PLT (× 10^9^/L)	278.8 ± 118.7	253.1 ± 145.7	0.337

## Discussion

Artificial intelligence is a challenging science comprising various fields and is increasingly employed in medical research, diagnosis, and treatment, particularly in medical imaging application research [15–17]. Notably, image datasets have been extensively employed to develop deep-learning diagnostic models for differentiating various diseases in visual imaging diagnosis [13, 18–20]. In various parts of the world, medical resources are limited, resulting in scenarios where patients in critical conditions suffering from lung infections could die due to a lack of timely treatment. Current approaches employed for pneumonia pathogen detection have several limitations, including low sensitivity and accuracy, prolonged waiting time, high labor costs, and the use of nonspecific drugs (such as broad-spectrum antibiotics), which could exacerbate the disease and increase hospitalization costs [21–23].

In this study, the MI-DenseCFNet multimodal diagnostic model was developed and validated. Based on a deep learning neural network, this model functions as an adjunctive diagnostic model, combining imaging histology with clinical features to effectively differentiate between SAP and ASP. First, the model’s comprehensive group diagnostic performance was assessed, indicating an AUC of 0.92, an F1 score of 0.875, and Kappa value of 0.75. The model’s classification diagnostic efficiency consistency was excellent. Compared with the deep diagnostic agent forest model developed by Chen et al [24], which yielded a combined AUC of 0.851 ± 0.003 in secondary pathogen identification, the accuracy of this model in separate ASP diagnosis analysis was 0.081 ± 0.005 and 0.781 ± 0.005 for SAP. Notably, our model demonstrated enhanced diagnostic accuracy. From a distinct perspective, it can also be demonstrated that obtaining better diagnostic accuracy is correlated with the inclusion of clinical features in our model. In addition, the color shade of the intensity of attention according to the heat map indicates that the model is better at extracting the imaging features.

In the external validation set, the model exhibited a diagnostic performance characterized by an AUC of 0.83, an F1 score of 0.846, and a Kappa value of 0.68, indicating a moderate level of classification and diagnostic efficiency agreement. The significant area under the ROC curve obtained in the external validation set demonstrates that this model can identify new chest CT images and clinical features that were not previously incorporated into the model. Moreover, a higher F1 score demonstrates that the model has good classification ability and no overfitting. However, a separate analysis of the model diagnostic accuracy for SAP and ASP indicated 89.9% vs. 61.1% for the imaging-only group, 90.3% vs. 84.7% for the combined group, and 93% vs. 74.8% for the external validation set. These findings demonstrate that the model exhibited reduced accuracy in diagnosing the ASP. This observation is associated with the characteristics of the included ASP images, as most of the ASP images are isolated nodules with a low number of lesion levels, leading to fewer data volume for the deep learning neural network in extracting image features. This difference can also be seen in the confusion matrix. Nonetheless, with the incorporation of clinical features, the model’s diagnostic accuracy demonstrated enhancement in the integrated group compared with the imaging-only group (88.1% vs. 78.4%). Previous investigations [25] demonstrated that their ML-based joint columnar maps exhibited good discrimination in the validation set for invasive fungal infections of the lung (AUC = 0.844), outperforming clinical (AUC = 0.696) and radiomics (AUC = 0.767) models. The overall performance of our model outperforms the results of this study, mainly because of the enhanced capability of deep learning convolutional neural networks in extracting image features compared with ML [26]. For invasive pulmonary aspergillosis (IPA), a study developed the IPA-NET diagnostic model, achieving an external validation set accuracy of 89.7% and an AUC of 0.95, thereby validating the model’s exceptional accuracy in diagnosing IPA [27]. However, the imaging features of IPA are significantly different from those associated with nonfungal pneumonia to some extent [28]. In our study, due to the challenges in collecting imaging data of ASP, some cases of putrefactive ASP attributed to tuberculosis and bronchiectasis were excluded. Furthermore, because of the more distinctive imaging features of ASP, the enrollment of chest CT of ASP can be expanded in future studies to enhance the diagnostic accuracy of ASP.

The advantage of our study is not only attributed to the incorporation of patients’ clinical information into our model but also to the identification of significant clinical features linked with SAP and ASP in our ML model. These identified features can be provided separately to clinicians in their routine clinical practice, serving as a criterion to determine whether a patient is infected with S. aureus or Aspergillus, thus offering empirical decisions for clinical purposes. In addition, in the following research, we can also screen out some clinical features that can optimize DNN extraction through the random forest classification diagnostic model, so as to further improve the accuracy of the MI-DenseCFNet multimodal diagnostic model. However, we find that the AUC of the validation set (95%) is much higher than the AUC of the training set (81.2%) in the random forest classification diagnostic model. We believe that the AUC of the training set is calculated when the model is trained, and the AUC of the validation set is calculated on the after-convergence training set model so that the AUC of the validation set is higher than the AUC of the training set [29]. In addition, because the amount of data brought in was not large enough, the training set and validation set data were grouped by a ratio of 8:2, which was exacerbated to some extent by the uneven distribution of data. We can increase the amount of data and adjust the proportion of the training set to improve the results. Furthermore, our distinct analyses of SAP and ASP studies enhance the credibility of our model in terms of diagnostic accuracy for these two types of pneumonia compared with previous studies [24]. Compared with the diagnostic performance of radiologists, the 20 cases of internal validation set data took only 10.24 s to arrive at a diagnosis in the imaging-only group model, with a diagnostic accuracy significantly higher than that of a junior radiologist with 2–3 years of experience (78% vs. 40%). Notably, the speed of the model to arrive at a diagnosis is dependent on computer hardware performance, which could achieve even faster speeds as technology continues to advance.

Our study has some limitations. The primary focus was to obtain consistently high-quality chest CT images; however, after strict screening using exclusion criteria, the total sample size of the enrolled group proved to be insufficient. Thus, the model encountered challenges in completely extracting the image feature vectors associated with SAP and ASP during the training process. Nonetheless, subsequently, we can let the model focus on learning about lesions by expanding the sample size or manually outlining the region of interest to further enhance the diagnostic accuracy of the model.

## Conclusion

The MI-DenseCFNet multimodal diagnosis model can effectively diagnose SAP and ASP, and its diagnostic performance significantly exceeds that of junior radiologists, thereby accelerating the diagnostic process of pathogenic diseases. The 11 important clinical features were screened in the constructed random forest dichotomous diagnostic model, providing a reference for clinicians.

### Supplementary information

ESM 1 (PDF 1091 kb)

## Abbreviations

ASP: *Aspergillus* pneumonia
AUC: Area under the curve
CNCB: China National Center for Bioinformation
CT: Computer tomography
DNN: Deep neural network
IPA: Invasive pulmonary aspergillosis
MI-DenseCFNet: Multi-input DenseNet fused with clinical features
ML: Machine learning
ROC: Receiver operator characteristic curve
SAP: *Staphylococcus aureus* pneumonia

## Declarations

### Guarantor

The scientific guarantor of this publication is Dr. Jianqing Zhang, who is the corresponding author of the article.

### Statistics and biometry

No complex statistical methods were necessary for this paper.

### Methodology

• retrospective

• multicenter study
