# Supplementary material for: MI-DenseCFNet: deep learning–based multimodal diagnosis models for Aureus and Aspergillus pneumonia
Source: Eur Radiol. 2024 Jan 17;34(8):5066–76. doi: 10.1007/s00330-023-10578-3 (PMC11254966; doi:10.1007/s00330-023-10578-3)

MI-DenseCFNet: Deep Learning-Based Multimodal Diagnosis Models for *Aureus* and *Aspergillus* Pneumonia

Electronic Supplementary Material (ESM)

Eur Radiol (2023) Liu T, Zhang ZH, Zhou QH et al

Supplemental 1. Model evaluation.

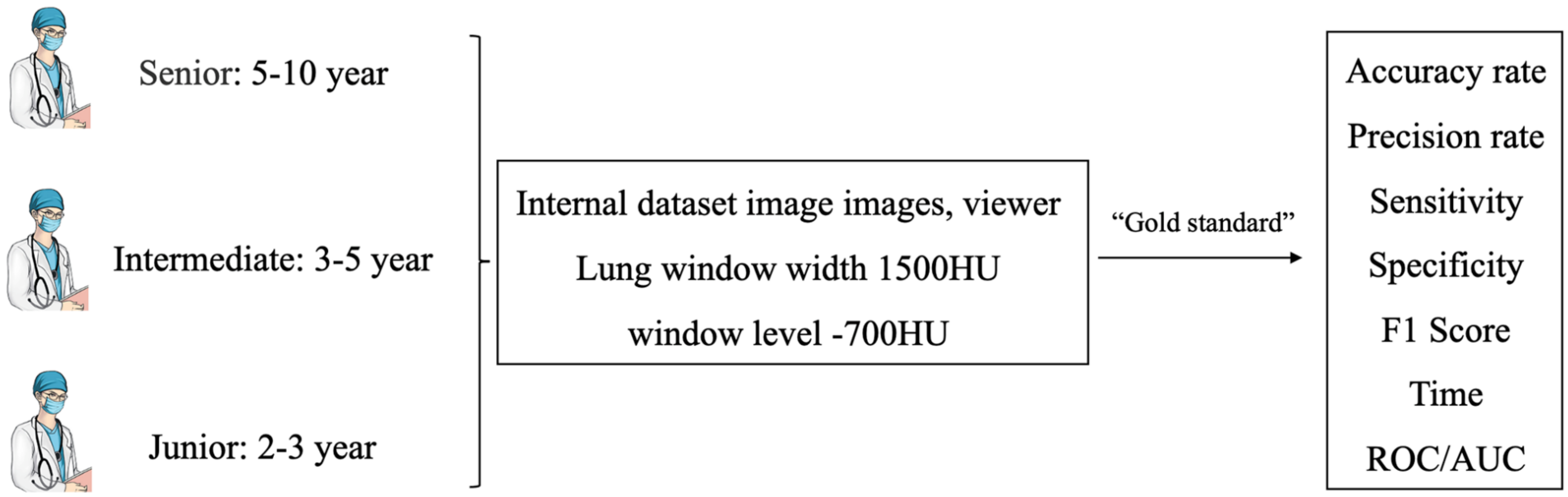

Supplemental 2. Performance of the deep learning diagnostic model.

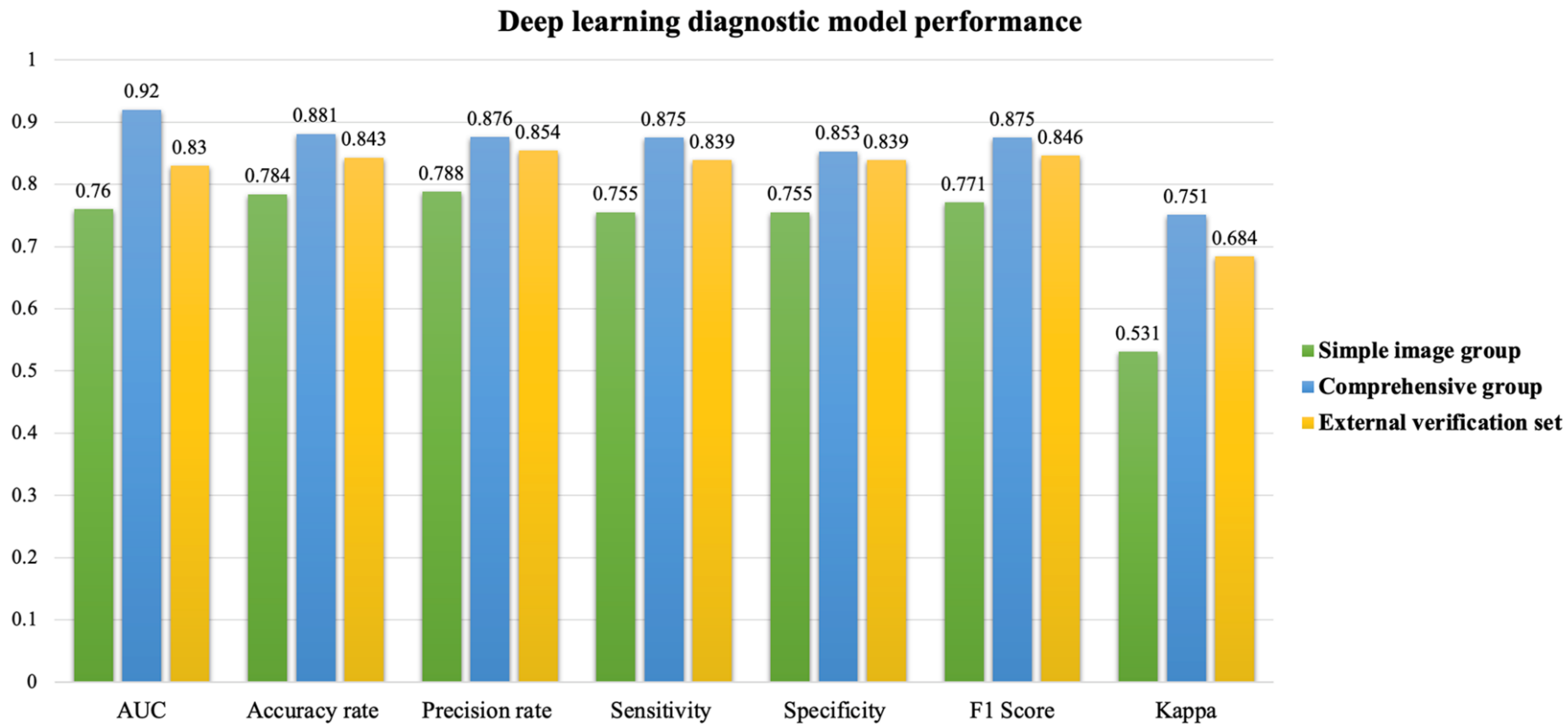

Supplemental 3. Comparison of model performance between radiologists with different years of experience and the imaging-only group.

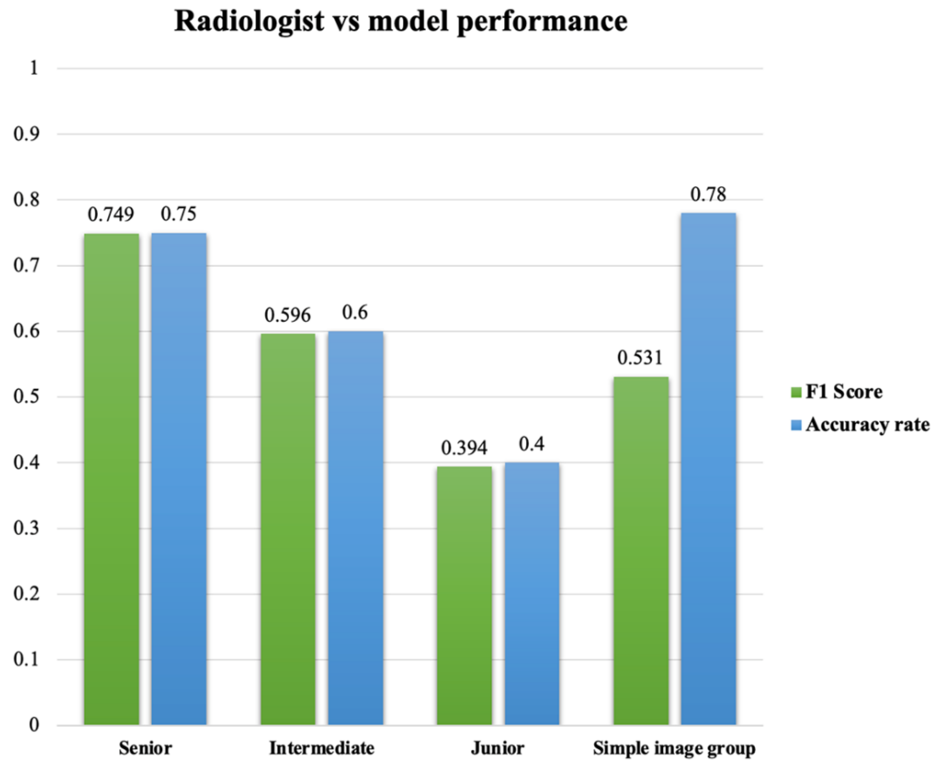

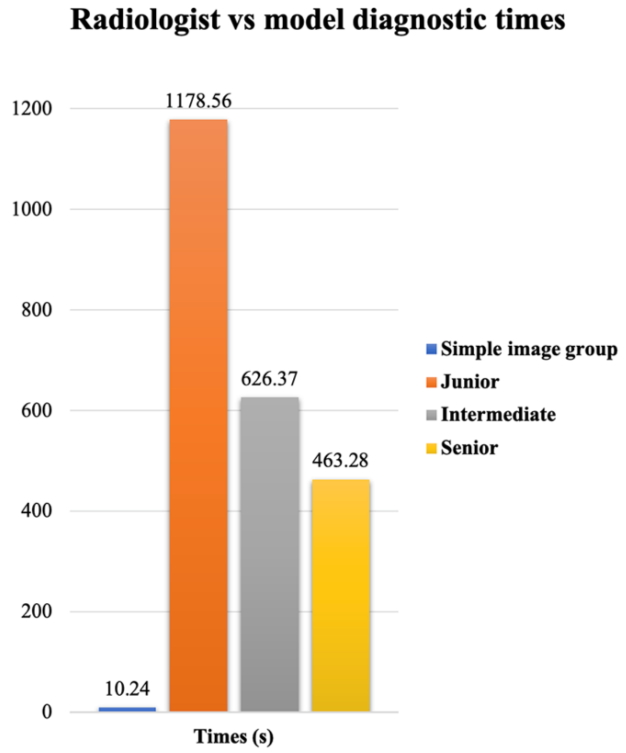

Supplemental 4. Top 20 model feature importance scores.

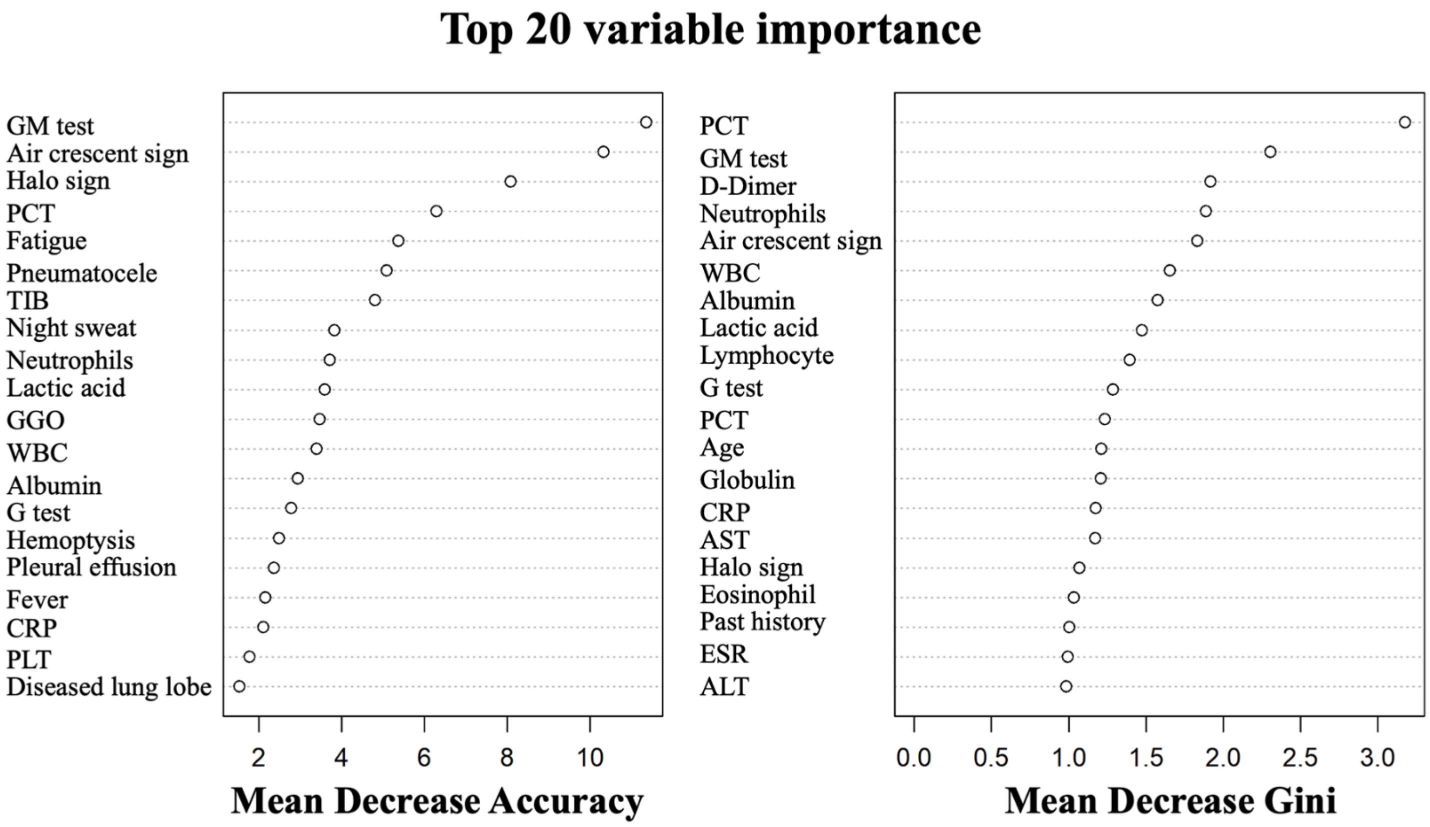

Supplemental 5. The left figure shows the training set ROC curve; the right figure shows the validation set ROC curve.

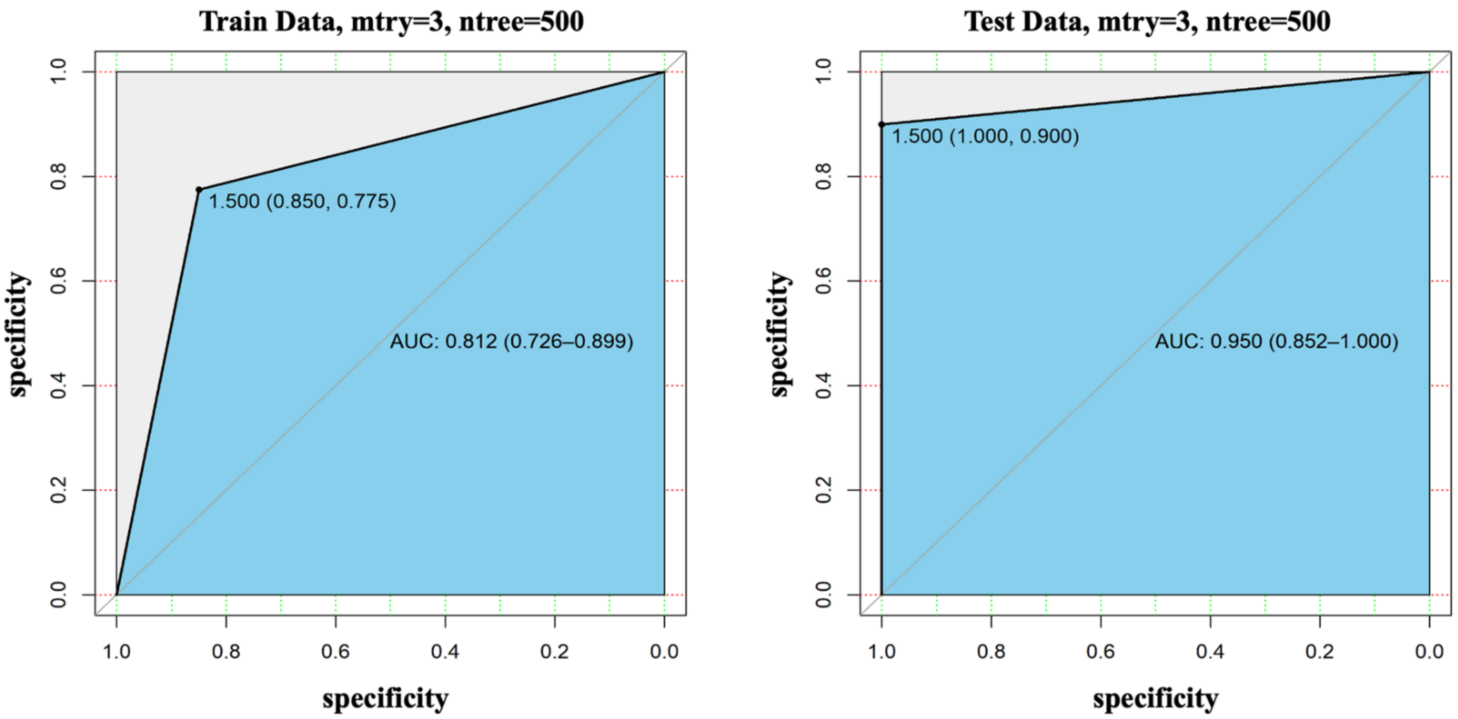

Supplement: Supplementary file 1 — (PDF 1091 kb) [file 330_2023_10578_MOESM1_ESM.pdf]
